# Can Sport Concussion Assessment Tool (SCAT) Symptom Scores Be Converted to Rivermead Post-concussion Symptoms Questionnaire (RPQ) Scores and Vice Versa? Findings From the Toronto Concussion Study

**DOI:** 10.3389/fspor.2021.737402

**Published:** 2021-10-22

**Authors:** Laura Kathleen Langer, Paul Comper, Lesley Ruttan, Cristina Saverino, Seyed Mohammad Alavinia, Elizabeth L. Inness, Alice Kam, David W. Lawrence, Alan Tam, Tharshini Chandra, Evan Foster, Mark T. Bayley

**Affiliations:** ^1^KITE Toronto Rehabilitation Institute, University Health Network Toronto, Toronto, ON, Canada; ^2^Toronto Rehabilitation Institute, University Health Network, Toronto, ON, Canada; ^3^Faculty of Kinesiology and Physical Education, University of Toronto, Toronto, ON, Canada; ^4^Rehabilitation Sciences Institute, University of Toronto, Toronto, ON, Canada; ^5^Graduate Department of Psychological Clinical Science, University of Toronto Scarborough, Toronto, ON, Canada; ^6^Toronto Western Hospital, University Health Network, Toronto, ON, Canada; ^7^Division of Physical Medicine and Rehab, Faculty of Medicine, University of Toronto, Toronto, ON, Canada; ^8^Department of Physical Therapy, University of Toronto, Toronto, ON, Canada

**Keywords:** concussion, Sport Concussion Assessment Tool (SCAT5), Rivermead Post-concussion Questionnaire, conversion equation, mild traumatic brain injuries, sports related concussion

## Abstract

**Background:** The Rivermead Post-Concussion Symptoms Questionnaire (RPQ) and the Sports Concussion Assessment Tool (SCAT) are widely used self-report tools assessing the type, number, and severity of concussion symptoms. There are overlapping symptoms and domains, though they are scored differently. The SCAT consists of 22 questions with a 7-point Likert scale for a total possible score 132. The RPQ has 16 questions and a 5-point Likert scale for a total of 64 possible points. Being able to convert between the two scores would facilitate comparison of results in the concussion literature.

**Objectives:** To develop equations to convert scores on the SCAT to the RPQ and vice versa.

**Methods:** Adults (17–85 years) diagnosed with a concussion at a referring emergency department were seen in the Hull-Ellis Concussion and Research Clinic, a rapid access concussion clinic at Toronto Rehab–University Health Network (UHN) Toronto Canada, within 7 days of injury. The RPQ and SCAT symptom checklists as well as demographic questionnaires were administered to all participants at Weeks 1, 2, 3, 4, 5, 6, 7, 8, 12, 16.

**Results:** 215 participants had 1,168 matched RPQ and SCAT assessments. Total scores of the RPQ and the SCAT had a rho = 0.91 (*p* < 0.001); correlations were lower for sub-scores of specific symptom domains (range 0.74–0.87, *p* < 0.001 for all domain comparisons). An equation was derived to calculate SCAT scores using the number and severity of symptoms on the RPQ. Estimated scores were within 3 points of the observed total score on the SCAT. A second equation was derived to calculate the RPQ from the proportion weighted total score of the SCAT. This equation estimated corresponding scores within 3 points of the observed score on the RPQ.

**Conclusions:** The RPQ and SCAT symptom checklists total scores are highly correlated and can be used to estimate the total score on the corresponding assessment. The symptom subdomains are also strongly correlated between the 2 scales however not as strongly correlated as the total score. The equations will enable researchers and clinicians to quickly convert between the scales and to directly compare concussion research findings.

## Introduction

Concussions, or mild traumatic brain injuries (mTBIs) are common injuries (Langer et al., 2021) that represent acute neurophysiological event related to blunt impact or other mechanical energy applied to the head, neck, or body (with transmitting forces to the brain), such as from sudden acceleration, deceleration, or rotational forces (Ontario Neurotrauma Foundation, 2018). The sequalae of symptoms associated with concussion can include headache, dizziness, nausea, sleep difficulties, irritability, difficulty concentrating, etc. (Willer and Leddy, 2006). Clinicians that treat patients with concussions need to assess their patients' number of symptoms, the systems affected, and the severity of symptoms to determine appropriate treatment(s) and to monitor recovery progression.

The Rivermead Post Concussion Symptoms Questionnaire (RPQ) was published in 1995 (King et al., 1995) as the first assessment of severity of post-concussion symptoms. It uses a 5-point Likert scale to determine the severity of 16 concussion related symptoms in somatic, cognitive, and emotional domains, with scores ranging from 0 to 64. It is also commonly used in concussion research in the general population. To date it has been cited in over 100 peer reviewed papers.

In 2005, the Sports and Concussion Group released the Sports Concussion Assessment Tool (SCAT), a field-side standardized tool to assess concussions in athletes 13 years and older (McCroy et al., 2005). It included a symptom evaluation checklist of 22 items in somatic, cognitive, and emotional domains and a 7-point Likert scale to determine symptom severity with scores ranging from 0 to 132. The SCAT is reviewed and updated every 4 years and is currently in the 5th iteration (McCrory et al., 2017). The SCAT is a common outcome measure in research in athletes with concussions and has been cited in over 200 peer reviewed papers.

There is considerable overlap between the RPQ and the SCAT in the domains assessed and specific symptoms such as headaches, sensitivity to noises and to lights, vision disturbances, nausea, irritability, sadness, fatigue, problems with concentration and memory, etc. The two scales are not directly comparable with different scoring for symptom severity and the SCAT having more emphasis on somatic symptoms and RPQ emphasizing cognitive symptoms. The objective of this study was to determine the degree of correlation between the 2 scales and derive equations that would facilitate easy conversion from the RPQ to the SCAT and vice versa.

## Methods

### Participants

The study was approved by the Research Ethics Board at the University Health Network (ID# 15-9214). Written informed consent was obtained from those willing to participate in the study.

Participants diagnosed with a concussion by a physician from emergency departments in one of 6 participating hospitals in Toronto, Canada between February 2016 and September 2017 were referred to the Hull-Ellis Concussion and Research Clinic for follow-up care within 1 week of injury. Participants were eligible if they had a Glasgow Coma Scale Score of 13–15, were between 17 and 85 years of age, were able to attend a first clinic visit within 7 days of their injury and provided written informed consent to study participation. Participants were excluded if they were involved in a motor vehicle accident, work-related injury, and if there were neurological findings on CT clinical imaging scans. Participants were assessed by a clinic physician at weeks 1, 2, 4, 6, and 8 or until deemed recovered by the clinic physicians. Research data collection involved baseline questionnaires such as demographics, health history, concussion symptoms, etc. and weekly follow-ups until week 8 and then at week 12 and 16 post injury. The RPQ and SCAT was administered to each participant at each research (Weeks 1, 2, 3, 4, 5, 6, 7, 8, 12, 16) visit with RPQ preceding the SCAT at each administration consistently.

### Assessments

#### Rivermead Post-concussion Symptoms Questionnaire (RPQ)

The RPQ consists of 16 symptoms with a 5-point Likert scale (0 = not experienced at all, 4 = a severe problem) that asked participants to compare their symptoms over the past 24 h to their pre-concussion symptom severity. Scores range from 0 to 64 points. The RPQ was administered on a computer using REDCap. There are two additional, optional non-specified self-disclosed symptom questions that are ranked on the same 5-point Likert scale, however the symptom(s) the patient included may not be related to their concussion.

#### Sport Concussion Assessment Tool (SCAT)

The SCAT symptom evaluation consists of 22 symptoms with a 7-point Likert Scale (0 = none, 6 = severe) and instructed participants to rate their current symptoms. Scores range from 0 to 132 points. The SCAT was administered on a computer using REDCap.

The two scales and the groupings by domain are shown in Table 1.

**Table 1:** Symptom domain groupings and the corresponding Rivermead Post-Concussion Questionnaire (RPQ) and Sports Concussion Assessment Tool (SCAT) symptoms.

**Symptom domain**	**RPQ symptoms**	**SCAT symptoms**
Somatic	Headache Dizziness Nausea/Vomiting Noise sensitivity Light sensitivity	Headache Pressure in head Neck pain Nausea/Vomiting Dizziness Balance problems Sensitivity to light Sensitivity to noise Fatigue or low energy
Cognitive	Forgetfulness, poor memory Poor concentration Taking longer to think	Feeling like “in a fog” Difficulty concentrating Difficulty remembering Confusion
Emotional	Being irritable, easily angered Feeling depressed or tearful Feeling frustrated or impatient	More emotional Irritability Sadness Nervous or anxious
Sleep	Sleep disturbance	Drowsiness Trouble falling asleep

### Statistical Analyses

All statistics were performed on SAS 9.4 (SAS Institute, North Carolina, USA). Descriptive analyses were performed for continuous and categorical variables for the entire sample and by week of administration (I, 2, 3, 4, 5, 6, 7, 8, 12, and 16). Spearman correlations were performed between the total score of the RPQ and SCAT, the number of symptoms for each measurement, subdomains of somatic symptoms (with and without fatigue included as a somatic symptom), emotional symptoms, sensory symptoms, and sleep related symptoms. Spearman correlations were also performed on the individual domains that measured the same symptom on each assessment (headache, dizziness, sensitivity to lights, sensitivity to sounds, fatigue, nausea, difficulty focusing, memory problems, difficulty falling asleep, and irritability) and “severity zones” based upon SCAT total score of low (0–25), moderate (26–75), and high (>76). An exploratory factor analysis was initially performed to determine which factors would be needed to derive an equation to convert a total score on the RPQ to the SCAT, however factors were too closely aligned to each other. A general linear selection model was then performed to identify key variables.

Identified key factors were used to create an equation that would convert RPQ total score to SCAT total score. The actual SCAT total score and the predicted SCAT score were compared to each other and numerous equations were tested until confidence interval of the predicted score was below ±5 points. A second equation using identified key factors was used to create an equation that would convert a SCAT score to an RPQ score. The actual RPQ score and the predicted RPQ score were compared for accuracy. The equation was refined until the confidence interval was less than ±5 points. Area under the curve (AUC) was calculated to determine the goodness of fit of each equation.

## Results

There were a total of 215 participants with 1,168 matched RPQ and SCAT assessments administered over the 16 weeks of this study. Females accounted for 60.4% of the cases. The mean age was 34.8 (SD 12.1) years. The mean overall SCAT total score was 30.0 (SD 28.6) out of 132 with a mean of 11.5 (SD 7.3) symptoms of a possible 22. The RPQ had an overall mean total score of 21.0 (SD 13.9) out of 64 and a mean of 10.2 (SD 5.0) symptoms of a possible 16. Demographics are presented in Table 2

**Table 2:** Demographics.

	**Mean (standard deviation)**	**Count (%)**
Males/Females		463 (39.6)/705 (60.4)
Age	34.8 (14.2)	
RPQ total score	21.0 (13.9)	
RPQ number of symptoms	10.2 (5.0)	
SCAT total score	30.0 (28.6)	
SCAT number of symptoms	11.5 (7.3)	
Week 1 Paired RPQ-SCAT		214 (18.3)
Week 2 Paired RPQ-SCAT		163 (14.0)
Week 3 Paired RPQ-SCAT		129 (11.0)
Week 4 Paired RPQ-SCAT		127 (10.9)
Week 5 Paired RPQ-SCAT		83 (7.1)
Week 6 Paired RPQ-SCAT		77 (6.6)
Week 7 Paired RPQ-SCAT		66 (5.7)
Week 8 Paired RPQ-SCAT		123 (10.5)
Week 12 Paired RPQ-SCAT		104 (8.9)
Week 16 Paired RPQ-SCAT		82 (7.0)

### Correlations Between SCAT and RPQ

#### Total Score and Symptom Number

**Figure 1:**
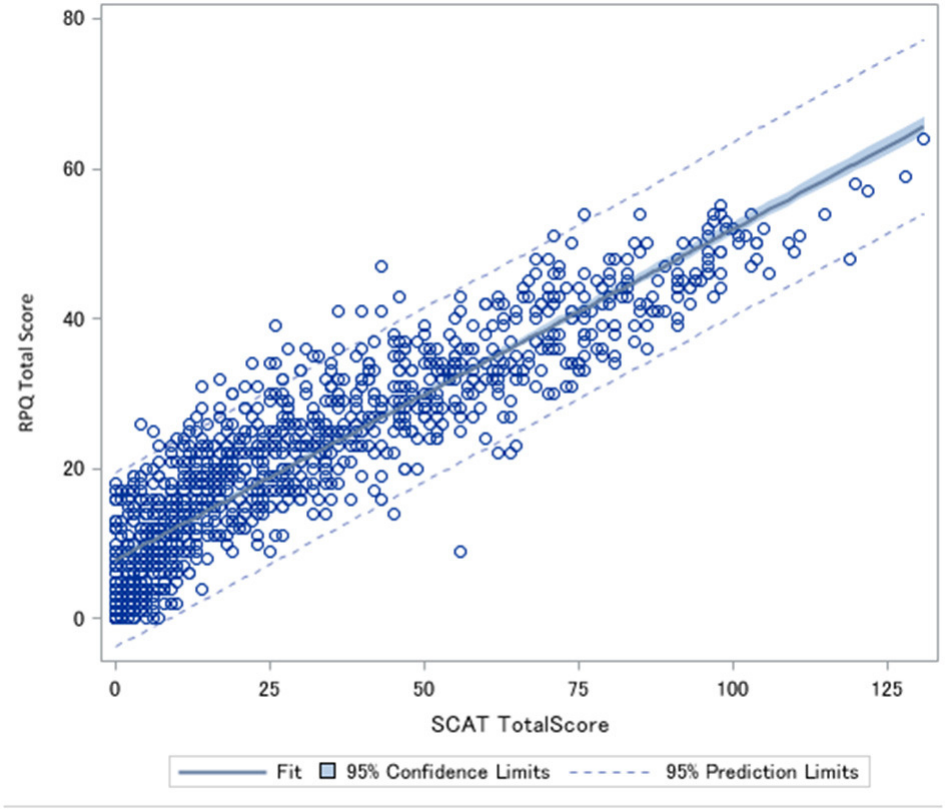
Spearman correlation between Rivermead post-Concussion Symptoms Score (RPQ) total score and Sports Concussion Assessment Tool (SCAT) total score. Rho = 0.91 *p* < 0.0001.

**Figure 2:**
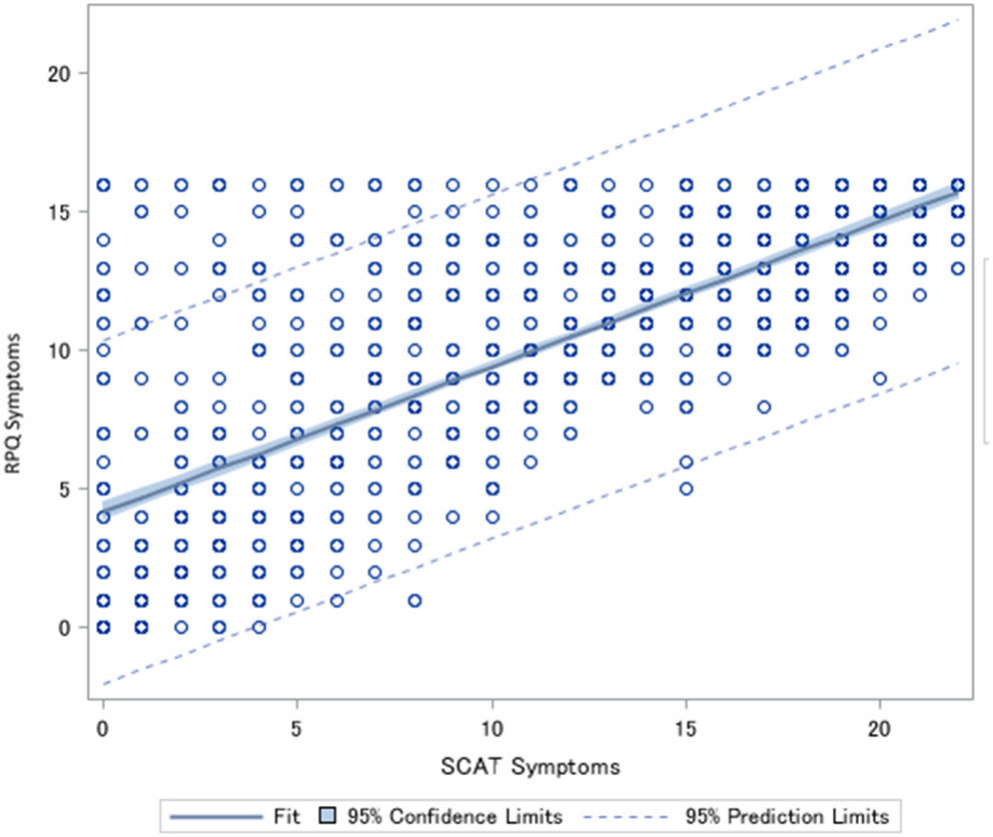
Spearman correlation between the number of symptoms endorsed on the Rivermead Post-Concussion Symptoms Questionnaire (RPQ) and the number of symptoms endorsed on the Sports Concussion Assessment Tool (SCAT). Rho = 0.77 *p* < 0.0001.

The Spearman correlation between the total scores of the SCAT and RPQ was 0.91 (*p* < 0.0001) (Figure 1) and the correlation between the number of symptoms was Rho = 0.77 (*p* < 0.0001) (Figure 2). Correlations between SCAT and RPQ total score by week are presented in Table 3. The correlation between the total scores on both measures remained very strong over time.

**Table 3:** Spearman correlations between the Rivermead Post-concussion Symptoms Questionnaire (RPQ) total score and the Sports Concussion Assessment Tool (SCAT) total score by week administered.

**Week**	**Spearman correlation (*p*)**
1	0.92 (<0.0001)
2	0.93 (<0.0001)
3	0.90 (<0.0001)
4	0.87 (<0.0001)
5	0.91 (<0.0001)
6	0.87 (<0.0001)
7	0.89 (<0.0001)
8	0.90 (<0.0001)
12	0.83 (<0.0001)
16	0.83 (<0.0001)

#### Domain Groupings

The RPQ and SCAT symptoms can be grouped into Somatic, Cognitive, and Emotional Symptom Domains (Potter et al., 2006; Asken et al., 2017). The correlation between the somatic symptom groups of the SCAT and RPQ was rho = 0.88 (*P* < 0.0001) and when fatigue was not included as a somatic symptom, the groups' correlation was rho = 0.87 (*p* < 0.0001). The Cognitive symptoms groups had a correlation of 0.83 (*p* < 0.0001), the Emotional symptoms groups had a correlation of rho = 0.85 (*p* < 0.0001), and the Sleep symptoms groups had rho = 0.74 (*p* < 0.0001). The symptoms included in each domain grouping is presented in Table 1.

#### Individual Domains

There were 11 individual symptom domains that were common to both measurements: headache (rho = 0.81, *p* < 0.0001), nausea (rho = 0.77, *p* < 0.0001), dizziness (rho = 0.78, *p* < 0.0001), sensitivity to noise (rho = 0.82, *p* < 0.0001) and light (rho = 0.87, *p* < 0.0001), blurred vision (rho = 0.73, *p* < 0.0001), fatigue (rho = 0.82, *p* < 0.0001), difficulty focusing (rho = 0.83, *p* < 0.0001), memory problems (rho = 0.81, *p* < 0.0001), irritability (rho = 0.82, *p* < 0.0001), and difficulty falling asleep (rho = 0.78, *p* < 0.0001).

#### SCAT Severity Zones

There were significant (*p* < 0.0001) moderate to strong correlations between the SCAT and RPQ based upon severity zones (Table 4), low (0–25 points), moderate (26–75), and high (>76) of the SCAT. The moderate range had the weakest (rho = 0.67), the low range had the strongest of the three ranges (rho = 0.75), and the high range had a ranked correlation of 0.70.

**Table 4:** Spearman correlations between Rivermead Post-concussion Symptoms Questionnaire (RPQ) and low (0–25), medium (26–75), and high (75–132) score zones on the Sports Concussion Assessment Tool (SCAT).

	**RPQ**		
**SCAT score zone**	***N***	**Spearman correlation**	***P***
Low	647	0.75	<0.0001
Medium	420	0.67	<0.0001
High	112	0.70	<0.0001

### Key Factors

Correlations became stronger as the domains were compressed, the domain groupings, total number of symptoms, and total score, as well as age and sex, were entered into an exploratory factor analysis to determine which were the key factors to retain to create a conversion equation from the RPQ to the SCAT. Communality was >1. A general linear selection model was then performed with the same factors. Four factors were retained: RPQ total score, RPQ symptom score, Cognitive domain group, and Sleep domain group. The Sleep domain group was removed on the second step-wise step. No other factors were retained.

### RPQ to SCAT Conversion Equation

**Figure 3:**
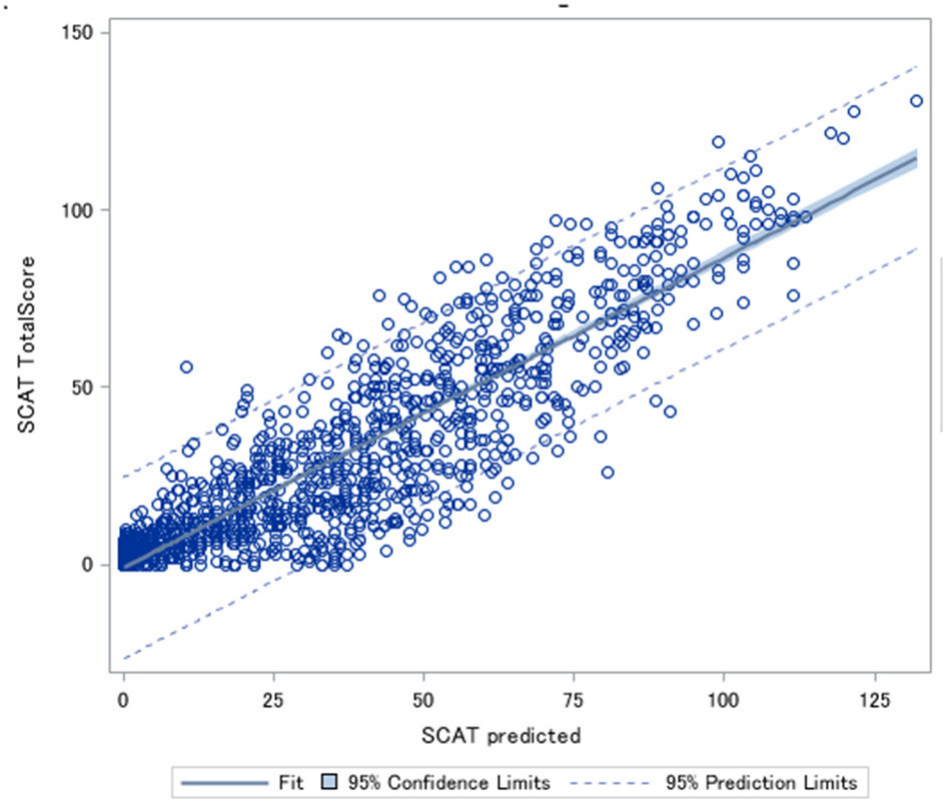
Predicted Sports Concussion Assessment Tool (SCAT) scores plotted against the actual SCAT scores.

An initial direct proportion equation was derived (RPQ total score/80)^*^132 however accuracy was poor and predictions were within 7 points in either direction of the actual score on the SCAT. A second equation that took into account weights of the domain groups was then tested (SCAT score = [(RPQ somatic domain score/max possible RPQ somatic score)^*^SCAT max possible somatic domain score] + [(RPQ Cognitive domain score/max possible Rivermead cognitive domain score)^*^max possible SCAT cognitive domain score] + [(RPQ emotional domain score/max possible RPQ emotional domain score)^*^max possible SCAT emotional domain score] + [(RPQ sleep domain score/max possible RPQ sleep domain score)^*^max possible SCAT sleep domain score)], however this had lower accuracy than the first conversion equation and had a confidence interval of 9 points in either direction of the actual SCAT score. In both cases, the prediction was more likely to over predict low scores and under predict higher scores. The equation derived (Equation 1) generated predictions that were within a confidence interval of ±2.98 points of the actual SCAT score (Figure 3). There was an Area Under the Curve (AUC) of 0.89 for this equation.


$$\begin{array}{l} SCAT=\left(7\left(\left(RPQ\frac{Total}{16}\right)/5\right)\right)\times \left(\left(RPQ\frac{Symptoms}{16}\right)22\right) \end{array}$$

Equation 1 Conversion of RPQ scores to SCAT scores

An example of converting from the RPQ to the SCAT with a RPQ total score of 33 and a symptom score of 14 ((7((33/16)/5))^*^((14/16)^*^22) = 55.7) would result in a SCAT score of 55.7, rounded to 56 and actual SCAT score range 54–62.

### SCAT to RPQ Conversion Equation

**Figure 4:**
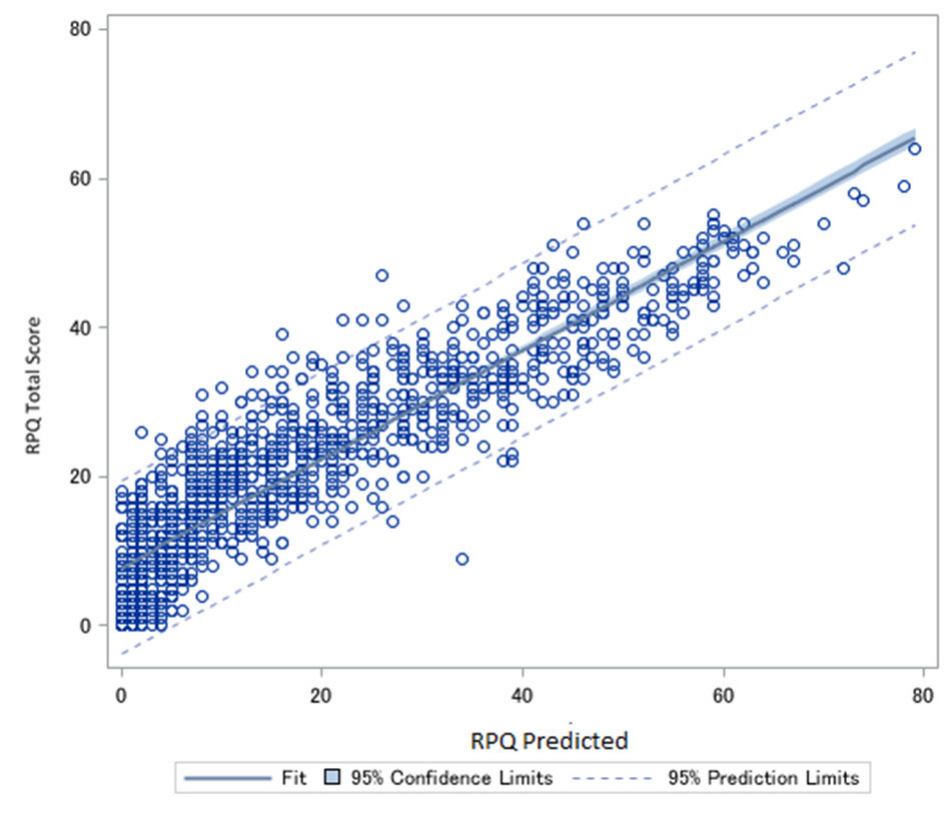
Predicted Rivermead post-concussion Symptoms Questionnaire (RPQ) scores plotted against actual RPQ scores.

A similar equation to the one that converted RPQ to SCAT using the mean intensity of the symptoms and the number of symptoms was derived to convert SCAT scores to the RPQ. Unfortunately, the confidence interval using this method was ±9.0 points. A conversion via direct proportions of the SCAT score (Equation 2) produced predictions within a confidence interval of ±3.1 points of the actual RPQ score (Figure 4) The AUC was 0.88 for this equation.


$$\begin{array}{l} RPQ\;=\left(SCAT\frac{Total}{132}\right)\times 64 \end{array}$$

Equation 2 Conversion of SCAT scores to RPQ scores.

An example of a transformation from the SCAT to the RPQ with a patient with a SCAT total score of 45 [(45/132)^*^64 = 21.8] (would convert to a RPQ score rounded to 22, and actual RPQ scores ranged 20–32 points.

### Conversion of Domains and Symptom Groupings

Conversion between measures for domain groupings and individual common domains was attempted however the accuracy of the predications was low for individual domains (i.e., ±1.5–2.5 points on a 7 point Likert scale) and on domain groupings (i.e., ±4–7 points).

## Discussion

This is, to the authors' knowledge, the first time a set of equations to convert between the RPQ and SCAT and vice versa have been derived. The RPQ and SCAT were found to have a very high level of correlation (0.91) for the overall score and moderate to strong relationships for the symptom domains. This will enable direct comparisons of the two concussion symptom severity scales.

### Total Score vs. Subscore Groupings

The correlation was the strongest for the overall total score than any of the symptom domains. This may be due to the fact that specific symptoms included for each domain grouping differ slightly between RPQ and SCAT and are not necessarily a direct comparison of the symptoms. The total scores provide a holistic overview of the severity of the concussion and not specific symptoms. Some symptoms are vague such as “don't feel right” and don't fit easily into any particular symptom domain. The derived equations were more accurate at the overall total score level than at the domain weighted level and may reflect the slight discrepancies in included symptoms in the two scales.

Correlations were also not as strong when severity zones were analyzed than the overall total score for all 1,168 matched pairs. Biases introduced by not counterbalancing the administration of the two assessments might be more evident with the smaller number of matched pairs and the weaker correlation in the moderate range of the SCAT might be due to cognitive fatigue on the SCAT after being administered the RPQ first, potentially worsening moderate symptoms for the second assessment or not being able to understand the instructions as well for the SCAT.

### Clinical Implications

Clinicians that treat patients with concussions can use these equations to convert their patients' scores on one scale to their preferred symptom evaluation scale if multiple scales are used over the course of the patient's treatment. This will allow continuity of care and enable better symptom and symptom severity tracking and monitoring of the patient as their concussion symptoms progress.

### Research Implications

Both the RPQ and the SCAT are widely used in research though the SCAT is more common in research in sports related concussions than in the general population. Studies that use either the SCAT or RPQ can now be directly compared using these equations. This could facilitate analysis using retrospective data abstraction using either the RPQ or the SCAT and potentially meta-analyses in the future as more studies can be included.

### Convert the RPQ to the SCAT

Converting scores from the RPQ to the SCAT require weighing for the differing Likert scales used and the different number of symptoms included in the 2 assessments. The SCAT has 6 more symptoms than the RPQ, mostly in the somatic symptom domain. As the SCAT is a field assessment of concussion, players and team physicians/athletic trainers may be more sensitive to somatic symptoms and these may be the first symptoms to manifest after injury. Converted scores are within 3 points of the actual SCAT score using this equation, which is well-below the observed standard deviation associated with the SCAT (28.6 in these data) and therefore assumed to be accurate. Some participants had SCAT scores that were substantially higher than what the RPQ predicted their SCAT score to be; this may be due to the SCAT being administered after the RPQ, wording differences in the SCAT instructions (“how do you currently feel?” in the SCAT compared to “Rate your symptoms in the past 24 h” in the RPQ), or the Likert scale used in the SCAT having more options than the RPQ.

### Converting the SCAT to the RPQ

The equation that converts SCAT scores to the RPQ is simpler than the equation that converts RPQ to SCAT score. The SCAT has a more sensitive Likert scale and more symptoms queried than the RPQ. The wording of the SCAT at administration is also potentially less confusing than the RPQ as it asks about current symptoms and their severity. There is also an opinion that the RPQ may be imprecise at measuring concussion specific symptoms (Smith-Seemiller et al., 2003; Eyres et al., 2005; Sullivan and Garden, 2011). The scores calculated by this equation are within 3 points of the actual RPQ scores and also well within the observed standard deviation for the RPQ (13.9 in these data) and can be assumed to be accurate predictions.

### Model Fit

Both equations produce a value with confidence intervals of 3 points of the original score. In healthy adults without a concussion, a SCAT score of 9 total points is typical (Balasundaram et al., 2017; Downey et al., 2018). The range of 3 points of the observed score is below the baseline variability of these scales and can be assumed to be highly accurate conversions. The ROCs for both equations were also found to have high AUC and high sensitivity and specificities, further supporting the accuracy of the values calculated by the equations.

### Limitations

The authors acknowledge some limitations with this study. The RPQ was always administered before the SCAT and not in a counterbalanced method and this may have resulted in order effect bias and potentially decision fatigue in the responses on the SCAT. Future studies wishing to replicate this study should utilize a randomized counterbalanced design in administering the RPQ and the SCAT to reduce these potential biases. The population is a relatively highly educated urban-dwelling general adult population and as such the findings may not be completely generalizable, particularly to those with workplace or MVC related concussions or pediatric patients with concussion. Though highly correlated, the RPQ and SCAT scores distribution was not linear and more equations that take the non-linearity into consideration may produce even more accurate conversions.

## Conclusions

These equations allow for quick and accurate conversion between the RPQ and SCAT scores and vice versa. This will facilitate direct comparisons of the research when different assessments of concussion severity are used. Clinicians that treat patients with concussions can convert their patient's score to their preferred assessment if previously assessed using RPQ or SCAT.

## Data Availability Statement

The raw data supporting the conclusions of this article will be made available by the authors, without undue reservation.

## Ethics Statement

The studies involving human participants were reviewed and approved by University Health Network Research Ethics Board. The patients/participants provided their written informed consent to participate in this study.

## Author Contributions

LL: conceptualization, writing, analysis, equation derivation, figures, and literature search. PC: study conceptualization and editing. LR, EI, AK, DL, and AT: editing and data interpretation. CS: analyses and data interpretation. SA: editing, analyses, and data interpretation. TC: editing and study coordination. EF: data collection and literature search. MB: writing, study conceptualization, and literature search. All authors contributed to the article and approved the submitted version.

## Funding

This study was supported by the Toronto Rehab Foundation.

## Conflict of Interest

The authors declare that the research was conducted in the absence of any commercial or financial relationships that could be construed as a potential conflict of interest.

## Publisher's Note

All claims expressed in this article are solely those of the authors and do not necessarily represent those of their affiliated organizations, or those of the publisher, the editors and the reviewers. Any product that may be evaluated in this article, or claim that may be made by its manufacturer, is not guaranteed or endorsed by the publisher.
